# Safety and efficacy of an intra-oral electrostimulator for the relief of dry mouth in patients with chronic graft versus host disease: Case Series

**DOI:** 10.4317/medoral.19429

**Published:** 2013-10-13

**Authors:** Yehuda Zadik, Itai Zeevi, Noa Luboshitz-Shon, Nasri Dakwar, Andy Wolff, Michael Y. Shapira, Reuven Or, Sharon Elad

**Affiliations:** 1 Chief Dental Officer, Israeli Air Force Surgeon General Headquarters, Israel Defense Forces Medical Corps, Tel Hashomer, and Attending, Department of Oral Medicine, Hebrew University-Hadassah School of Dental Medicine, Jerusalem, Israel; 2 Resident, Department of Oral and Maxillofacial Surgery, Rabin Medical Center, Beilinson Campus, Petach-Tikva, Israel. At the time of the study: Department of Oral Medicine, Hebrew University – Hadassah School of Dental Medicine, Jerusalem, Israel; 3 Instructor, Department of Prosthodontics, Hebrew University – Hadassah School of Dental Medicine, Jerusalem, Israel; 4 Student, Dentist, private dental office, Tarshiha, Israel: Department of Oral Medicine, Hebrew University -Hadassah School of Dental Medicine, Jerusalem, Israel; 5 Chief Medical Officer, Saliwell Ltd., Harutzim, Israel; 6 Associate professor, Department of Bone Marrow Transplantation and Cancer Immunology, Hadassah University Medical Center, Jerusalem, Israel; 7 Professor-Department of Bone Marrow Transplantation and Cancer Immunology, Hadassah University Medical Center, Jerusalem, Israel; 8 Professor, Division of Oral Medicine, Eastman Institute for Oral Health, Rochester, USA. At the time of the study: Department of Oral Medicine, Hebrew University – Hadassah School of Dental Medicine, Jerusalem, Israel

## Abstract

Objectives: Patients with chronic graft-versus-host disease (cGVHD) often suffer from dry mouth and oral mucosal lesions. The primary objective of this study was to investigate the safety of an intra-oral electrostimulator (GenNarino) in symptomatic cGVHD patients. The secondary objective was to study the impact on the salivary gland involvement of cGVHD patients. Study Design: This paper presents a case series. The study included patients treated for 4 weeks, randomly assigned to the active device and then crossed-over to a sham-device or vice versa. The patients and clinicians were blind to the treatment delivered. Data regarding oral mucosal and salivary gland involvement were collected. Results: Six patients were included in this series. Most of the intraoral areas with manifestations of cGVHD were not in contact with the GenNarino device. Two patients developed mild mucosal lesions in areas in contact with the GenNarino during the study. However, only one of them had a change in the National Institutes of Health (NIH) score for oral cGVHD. The unstimulated and stimulated salivary flow rate increased in 4 out of the 5 patients included in this analysis. Symptoms of dry mouth and general oral comfort improved. Conclusion: This study suggests that GenNarino is safe in cGVHD patients with respect to oral tissues. Furthermore the use of GenNarino resulted in subjective and objective improvements in dry mouth symptoms. A large scale study is needed to confirm the impact and safety of GenNarino on systemic cGVHD.

** Key words:**Dry mouth, graft versus host disease, electrostimulation, oral mucosa, hematopoietic stem cell transplantation.

## Introduction

Chronic graft-versus-host disease (cGVHD) is a serious complication of allogeneic hematopoietic stem cell transplantation (HSCT). cGVHD is an alloimmune inflammatory process, which results from a donor-origin cellular response to host tissues (1). cGVHD damages both the oral mucosa and the salivary glands. Salivary gland involvement in cGVHD is characterized by reduced salivary secretion and xerostomia (2-4). Common areas of mucosal cGVHD involvement are the tongue, buccal and labial mucosae (5,6). The clinical and histopathological presentations of salivary cGVHD resemble the changes seen in Sjögren`s syndrome (1).

Oral moisturizing agents and systemic sialogogues help relieve xerostomia. However, the topical preparations only provide transient relief and therefore require frequent applications, whereas the systemic agents have side effects (7). The search for an optimal treatment modality continues.

An intra-oral electro-stimulating device named GenNarino has been introduced recently (8-10). The device aims to increase saliva secretion by stimulating fibers of the cranial nerves V and VII in the region posterior to the lower 3rd molar (10,11). Two multi-center trials showed decreased xerostomia and significant improvements in subjective dry mouth symptoms. No significant adverse events were reported. Oral mucosal lesions that could be related to the use of the device were observed at one follow-up visit in a few patients, but adjustment of the device yielded immediate resolution of the lesions (9,10).

Most of the reports on GenNarino refer to its use in patients suffering from dry mouth due to Sjögren`s syndrome, radiation induced salivary gland damage or drug-induced hyposalivation. Although all these patient populations are similar to GVHD patients regarding signs and symptoms of dry mouth, the underlying characteristics of GVHD patients raise important safety concerns, specifically:

1. In cGVHD patients the oral mucosa may be severely involved with areas of erythema, lichenoid, ulceration or mucoceles. cGVHD symptoms may deteriorate when the patient experiences stress of any nature e.g. emotional or physical stress due to mechanical injury which the device may cause.

2. Patients with cGVHD have a fragile immune system, causing fluctuations in their general health status. These patients are hospitalized repeatedly due to systemic infections and their complications. Potentially, the incidental peaks of systemic illness may override the baseline effects of local electrostimulation. Hypothetically, the intra-oral electrostimulator may cause stress to the patient and trigger or exacerbate systemic symptomatic cGVHD.

In this study we will describe the effect of the intra-oral electrostimulator in a series of oral cGVHD patients. The primary objective was to assess the effects on the oral mucosa (safety) and the secondary objective was to study the impact on the salivary gland involvement of cGVHD patients (efficacy). Part of the data for some of the patients reported here were included in a previous article about a multi-center study (9). The sample described in this study includes additional patients. The response of the oral mucosa in cGVHD patients has not been previously reported.

## Patient and Methods

Device description

**Figure 1:**
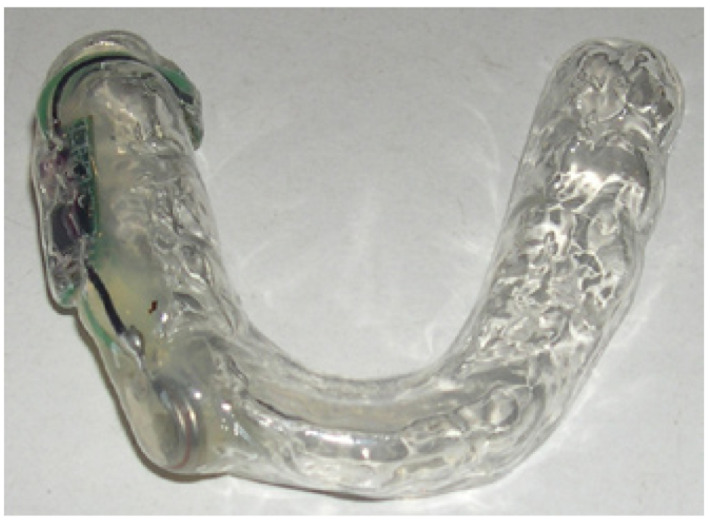
The device is fabricated individually to lie on the lower dentition loosely. The electric circuit is embedded in the acrylic body. The stimulating electrodes are positioned in the area of the mandibular third molar, in proximity to the lingual nerve.

The intraoral electrostimulation device consists of a mouth piece made to fit the mandibular dental arch and an infrared remote control (Fig. 1). It contains an electronic circuit (with a microprocessor and a receiver of remote control signals), a pair of stimulating electrodes, and a battery. The electrodes directly contact the oral mucosa in the mandibular third molar area, in proximity to the lingual nerve; therefore no conductive gel is needed. The patient activates and deactivates the electrical stimulation by pressing the “on” and “off” buttons on the remote control, respectively. The intensity of the electrical current is so low that it is not felt by the patient. An amber light on the intra-oral device blinks upon activation of the remote control to show that the device received the signal from the remote; devices were replaced if the light did not blink. The amber light responded in the same way in the sham remotes.

Study design

A series of 6 oral cGVHD patients is presented. Generally, the study design was a sham-controlled, crossover randomized, double blind study. It was approved by the hospital ethics committee and conducted in accordance with the Declaration of Helsinki. At the baseline visit all participants signed an informed consent form, demographic data and medical background were recorded, patients were evaluated clinically (see Clinical Evaluation), and impressions for custom made GenNarino devices were taken. The patients were provided with one GenNarino device, and two identical remote controls, one that switched GenNarino on and off (active arm), and a second that had no effect (sham arm). Each remote control was used for four weeks in an order determined by a randomized schedule. In the active state the device emitted electrical stimuli which were below the threshold of tactile sense, while in the sham state the device did not emit any electrical stimuli. In both arms the foreign body, i.e. the device, exerted mechanical stimuli in the oral cavity (see Device Description below). The device was delivered after baseline measurements were taken. On visit 2, one month later, the remotes were swapped. Visit 3 took place one month later. The patients and the investigators were blind regarding the type of the remote control (active/sham). Safety analysis included data from all patients for all visits.

Due to the fact that very few patients were recruited, the comparative aspect of the study was reduced and data are presented as a case-series. The data referring to oral mucosal response to the intra-oral electrostimulator and the safety are very important and have been summarized.

Inclusion and exclusion criteria

Subjects were included if they were post allogeneic HSCT, had cGVHD and xerostomia and signed the informed consent form. Patients were excluded if they met the following criteria: 1) younger than 18 years old; 2) seropositive for human immunodeficiency virus or hepatitis C virus; 3) wearing pacemakers or defibrillators; 4) allergy to the materials used in the electrostimulation device; 5) mental disease or depression; 6) pregnancy; 7) chronic or recurrent, erosive or ulcerative, or premalignant or malignant oral lesions (except for oral cGVHD); 8) oral anatomic characteristics precluding the use of the device; and 9) edentulous condition dental implants that could support the GenNarino would considered equivalent to teeth. Patients taking bisphosphonate drugs before and during the trial were excluded due to the unknown risk of osteonecrosis of the jaws following electrostimulation.

Patients taking systemic sialagogues were asked to discontinue their medication during the trial. Dosage of systemic immunosuppressive medications was unchanged during the study period in order to avoid potential alterations in salivary flow rates.

Clinical evaluation

At all three visits a clinical examination was preformed, questionnaires were administered, and safety-related information was obtained.

The clinical examination included scoring oral cGVHD according to the NIH scale (12). Analysis referred to the total severity score as well as to the type of cGVHD-related oral manifestations present (lichenoid, erythema, ulceration and mucocele) and the cGVHD distribution (lip, labial mucosa, buccal mucosa, tongue and soft palate). In addition cGVHD signs were categorized according to the surfaces in contact with the GenNarino (oral mucosa not in contact with the GenNarino, oral mucosa in contact with the GenNarino except the electrodes, oral mucosa in contact with the electrodes). Oral mucosal lesions which were not attributed to cGVHD were also documented. The oral evaluation included sialometry. Unstimulated sialometry was conducted for 5 minutes while the patient was at rest. For stimulated sialometry, the patient chewed a standard piece of parafilm for 10 minutes, and saliva was collected during the last five minutes of chewing. Saliva volume was determined gravimetrically (13).

The patients filled in a questionnaire about symptoms related to dry mouth and quality of life. Questions were about the level of “dry mouth”, “mouth comfort”, “difficulty while speaking”, “difficulty while swallowing”, and “quality of life” (QoL). Answers were reported using 100-mm visual analog scales (VAS) from the worst on the left to the best on the right.

The safety-related outcome measures included vital signs and changes in general health, which were assessed at each visit. Any discomfort caused by the electrostimulation device and any adjustments needed were recorded.

Statistical analysis

Due to the small sample size, only descriptive statistics were performed. The rate of positive events (i.e. findings compatible with oral cGVHD) was calculated.

## Results

Patients 

Nine patients were screened. Two were excluded due to bisphosphonate treatment and one was excluded because they were unable to attend all the requisite visits. The medical history and basic efficacy parameters are described for each of the 6 cGVHD patients in the study followed by detailed descriptions of the primary outcomes of this study, namely, the safety parameters related to oral cGVHD.

Case 1

A 31-year-old male with a history of Non-Hodgkin Lymphoma (NHL), conditioned with fludarabine, busulfan and total body irradiation following HSCT (reduced intensity protocol), developed cGVHD including a complaint of dry mouth. At 57 months after HSCT he was enrolled in the study. Before using the electrostimulator his only medication was oxazepam.

After GenNarino use the oral mucosal cGVHD lesions were only on surfaces not in contact with the device. The NIH score increased slightly after the active treatment ( Table 1).

**Table 1:**
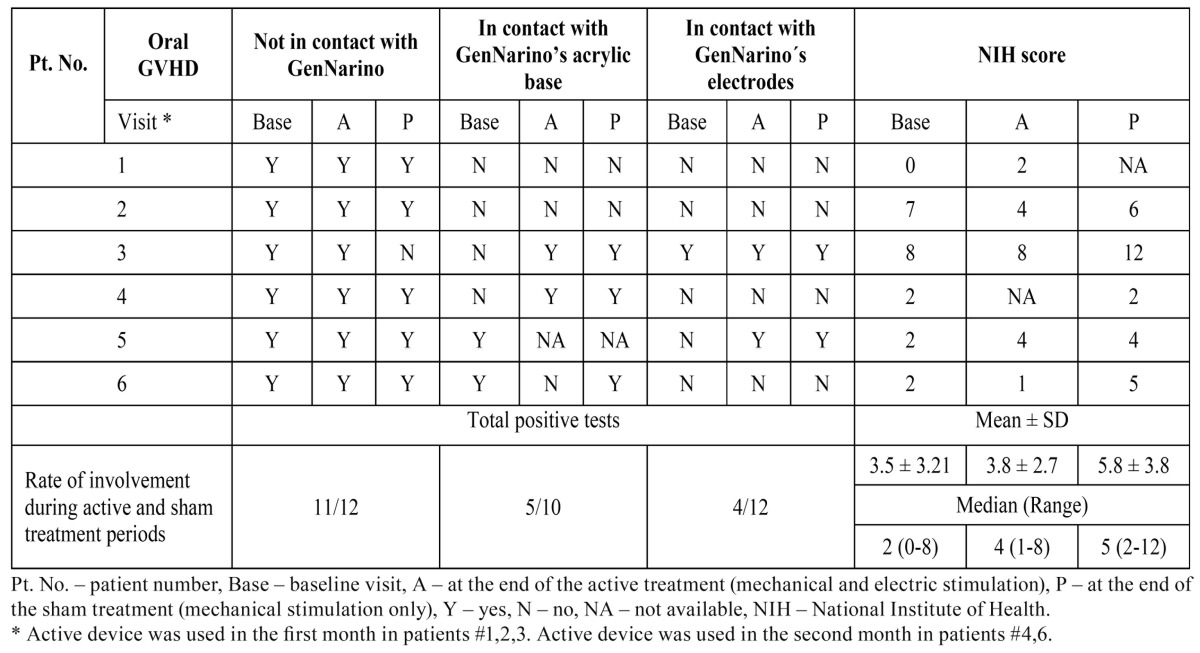
Mucosal Oral cGVHD manifestations per visit.

The patient’s score for “dry mouth” and “general mouth comfort” improved after application of the GenNarino, with better results from the active device ( Table 2). The categories “difficulty while speaking”, “difficulty while swallowing” and QoL did not improve.

**Table 2:**
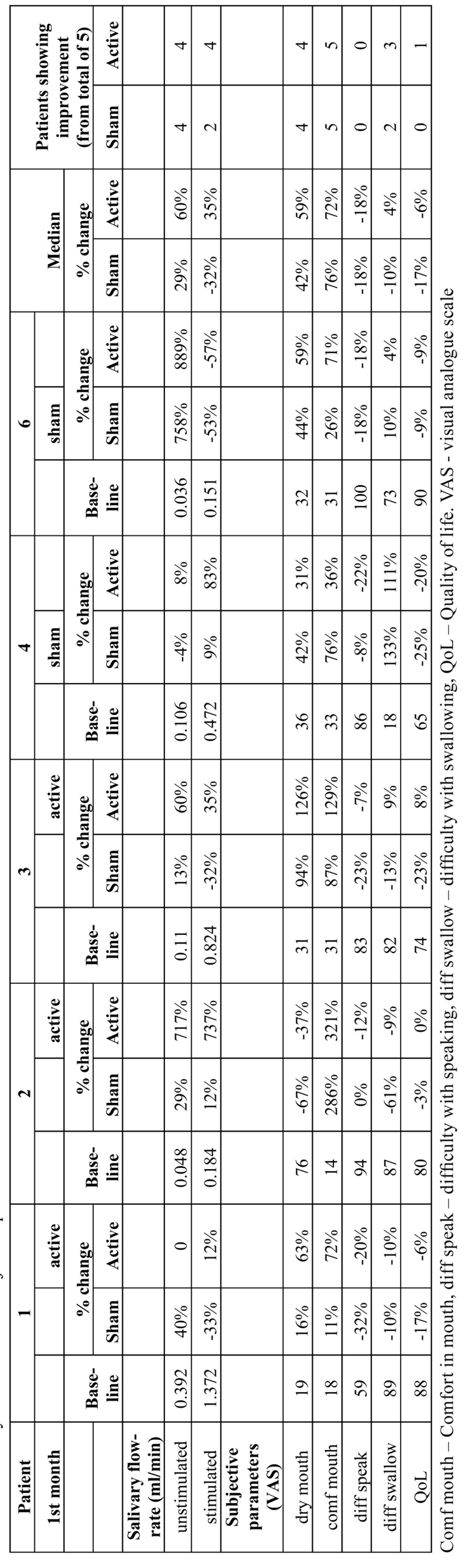
Salivary flow rates and subjective parameters.

Sialometry had mixed results ( Table 2). Baseline readings were 0.392 and 1.372 ml/min for unstimulated and stimulated sialometry, respectively. After the active device was used, the unstimulated salivary flow was unchanged and the stimulated sialometry increased by 12% compared to baseline. After the sham treatment in the second month the unstimulated secretion increased in 40% and the stimulated secretion decreased 33% compared to baseline.

Case 2

A 68-year-old man with a history of hypertension and Acute Myelogenous Leukemia (AML) for which he underwent allogeneic HSCT was referred for experimental treatment with GenNarino. The conditioning for the HSCT was of reduced intensity and included fludarabine and busulfan. Following HSCT he developed cGVHD including symptomatic salivary gland involvement. At nine months following the HSCT he started treatment with the GenNarino. At the start of the protocol his medications included cyclosporine A, zolpedim, metopropol, acyclovir, cilazapril, and sulfametaxazole.

Oral mucosal cGVHD involvement was only on surfaces not in contact with the device. The NIH score decreased with time, more so after the active device was used.

According to the patient’s questionnaire, “general mouth comfort” improved ( Table 2). Other questions did not show subjective improvement.

At baseline the unstimulated and stimulated salivary flow were 0.048 and 0.184 ml/min, respectively ( Table 2). After the sham treatment, during the second month the unstimulated secretion increased by 29% and stimulated secretion increased by 12% compared to the baseline. Following use of the active device the unstimulated salivary increased by 717% and the stimulated sialometry increased by 737% compared to the baseline. The patient did not use any saliva substitutes or saliva stimulants (chewing gum) during this period.

Case 3

A 66-year-old man presented with a complaint of dry mouth 8 months after allogeneic HSCT. His medical history included AML. The reduced-intensity conditioning for the HSCT included fludarabine and busulfan. He had no other co-morbidities. At the beginning of the study his medications were omeprazole, miodarone hydrochloride, tacrolimus, acyclovir, folic acid, warfarin, vitamin B, magnesium, imunoglubolin normal human, and sulphamethoxazole/trimethoprim.

The oral mucosal cGVHD was relatively severe at baseline with a NIH score of 8. Oral cGVHD did not change after active treatment and deteriorated after the sham treatment ( Table 1). This deterioration was part of an episode of severe systemic cGVHD (see Safety Parameters).

Four out of the 5 subjective parameters improved after the active treatment, including “dry mouth”, “general mouth comfort”, “difficulty while swallowing” and QoL ( Table 2).

At baseline the unstimulated and stimulated salivary flow were 0.11 and 0.824 ml/min, respectively ( Table 2). After active treatment unstimulated salivary flow rate increased by 60% and the stimulated sialometry increased by 35% compared to baseline. After the sham treatment during the second month unstimulated secretion increased by 13% and stimulated secretion decreased by 32%.

Case 4

A 51-year-old man with a history of hypertension and NHL, for which he underwent allogeneic HSCT, was referred 34 months post-HSCT with a complaint of dry mouth. The reduced-intensity conditioning for the HSCT included fludarabine, busulfan and ATG. He developed cGVHD and was treated with cyclosporine A, ramipril, omeprazole, prednisolone, dapsone, acyclovir, fluconazole, ursodeoxycholic acid.

The oral cGVHD score seemed to be stable before and after the sham treatment with GenNarino. Lesions appeared in areas in contact with the device ( Table 1).

Three subjective parameters improved, including “dry mouth”, “general mouth comfort” and “difficulty while swallowing” ( Table 2). The improvement was greater after sham treatment than after active treatment.

At baseline the unstimulated and stimulated salivary flow were 0.106 and 0.472 ml/min, respectively ( Table 2). The sham treatment was used first. After the sham treatment the unstimulated secretion decreased by 4% and the stimulated secretion increased by 9%. After active treatment the unstimulated and stimulated salivary increased by 8% and 83%, respectively.

Case 5

A 36 year-old man suffering from dry mouth was referred to our clinic. He was diagnosed with Acute Lymphoblastic Leukemia (ALL) for which he underwent allogeneic HSCT. Data about the conditioning regimen was not available as transplantation was carried out in a different country. His medical background included asthma and cGVHD. At the start of the study, 71 months after HSCT, he was medicated with omeprazole, prednisone and ursodeoxycholic acid.

When the randomization key was opened, we discovered that the randomization data of patient #5 was missing; therefore only the results for oral cGVHD, relevant to device safety were included. Sialometry data were not analyzed.

Generally the NIH score increased after the use of the electrostimulator. Oral mucosal cGVHD was present prior to the application of the GenNarino on surfaces not in contact with the device and appeared in areas in contact with the electrodes. The patient tolerated the treatment well.

Case 6

A 64-year-old female with a history of HTN, hypothyroidism and AML enrolled in the study. She underwent allogeneic HSCT 124 months prior to initiation of the GenNarino treatment. Upon initial examination medications were simvastatin, nifedipine, levothyroxine sodium, sulfametaxazole, prednisolone and pilocarpine hydrochloride. She stopped using pilocarpine in order to follow study protocols.

Oral mucosal cGVHD was mostly in areas not in contact with the GenNarino. The NIH score fluctuated during the periods of sham/active treatment.

Patient reported “dry mouth”, “general mouth comfort” and “difficulty while swallowing” to be better after active treatment ( Table 2).

At baseline the unstimulated and stimulated salivary flow were 0.036 and 1.514 ml/min, respectively ( Table 2). The sham treatment was used in the first month. After the sham treatment the unstimulated secretion increased by 758% and the stimulated secretion decreased by 53%. After the active treatment the unstimulated salivary flow increased by 889% and the stimulated salivary flow decreased by 57%. The patient did not use any saliva substitutes or saliva stimulants (chewing gum) during this period.

Oral mucosal cGVHD

Lichenoid was the most common type of lesion found in all patients at all visits. Erythema was the second most common type of manifestation presenting in 57.1% of visits in 3 patients. Ulceration was observed in 55.5% of the evaluations in 3 patients. Mucoceles were not observed.

The signs of oral mucosal cGVHD were mostly on the buccal mucosa (100% of patients), tongue, labial mucosa and lips (each in 20% of patients). The soft palate was only involved in one patient.

The most severe cGVHD score, based on the NIH scale, was observed following sham treatment ( Table 1).

Most of the oral manifestations of cGVHD were not on surfaces in contact with the GenNarino ( Table 1). Four patients had oral cGVHD signs in areas touching the GenNarino. Two of them developed these lesions during follow-up. The lowest rate of oral cGVHD signs was noted on the mucosal surface in contact with the electrodes presenting in 2 patients. There was no clear trend during the trial for increasing or decreasing cGVHD manifestations in areas in contact with the device. The only non-cGVHD oral lesion recorded during the study was gingivitis (patient #1). This patient had poor oral hygiene which did not improve despite repeated oral hygiene instruction.

Safety parameters

No patients complained about oral pain, feeling the electric stimulus or a burning sensation in the oral tissues. At the end of the trial, one patient (patient #1) reported that the device made him feel nauseous. It is worth mentioning that this patient suffers from dentophobia and has a very sensitive gag reflex that may be the cause of his complaint.

Two patients were hospitalized. These serious adverse events (SAE) were classified as not-related to the study-device. The first SAE (patient #5) was septic arthritis in the right knee and left elbow one week after the study ended, the patient complained of knee pain and swelling, he was afebrile during hospitalization. He was treated empirically with cefamezine and rocacefine which were switched to penbritin and gentamycin when group B streptococcus was isolated from blood and knee samples. His condition improved and he was referred for physiotherapy. The second SAE (patient #3) was a deterioration of systemic cGVHD, mostly in the liver, during the study. The patient was treated with prednisone, tacrolimus, ursodeoxycholic acid and supportive care and responded well. The patient did not continue to participate in the study due to this SAE.

## Discussion

cGVHD affects numerous target organs including the oral mucosa with fluctuating severity, depending on responses to specific triggers. Therefore, if an intraoral electrostimulator is to be used to relieve xerostomia among cGVHD patients, it must not exacerbate symptoms of oral mucosal and systemic cGVHD. Consequently, our main objective was to assess the effect of GenNarino on oral mucosal cGVHD and safety parameters. The results of this study show that GenNarino is safe. No severe adverse oral events were reported. After deleting the data of the patient that presented unstable systemic cGVHD, the NIH score for oral cGVHD at baseline and following the active treatment with the electrostimulator was similar. Furthermore, the distribution of oral manifestations of cGVHD was typical to the presentation of oral cGVHD that is reported in the literature with a slight increase in the rate of involvement in areas contacting the device (2 out of 5 patients developed mild involvement on new areas e.g. mild erythema). In other words, GenNarino did not trigger or aggravate oral cGVHD.

The systemic severe adverse events documented included infection and deterioration of systemic cGVHD. Infection is a common complication in post-HSCT patients because of their underlying immunosuppression. The cause of the deterioration in the systemic GVHD in second case of SAE was unclear; these episodes may arise due to emotional, physical or pathological triggers. This particular episode was serious, and the patient was given high dose steroids; during recovery, the complaint of dry mouth also resolved.

The efficacy of this treatment for dry mouth was suggested by the results of sialometry and subjective tests. Salivary flow rate increased in 4 out of 5 patients using the active device. All patients reported improvement in “dry mouth”, and oral “general comfort” levels and some improvement in “difficulty while swallowing”. However, no improvements were seen in other subjective parameters “difficulty while speaking” and general QoL. These findings were not surprising since numerous factors influence swallowing, speaking and general QoL, such as oro-pharyngeal mucosal status in cGVHD patients and concurrent systemic debilitating conditions. Unfortunately the patient sample was too small to calculate statistical significance. However, our descriptive findings support the literature regarding GenNarino for the treatment of drug induced dry mouth and Sjögren’s syndrome (8,14) .

According to the International Consensus organized by Germany, Austria and Switzerland (15) and the NIH Consensus Development Project on Criteria for Clinical Trials in cGVHD (16), cGVHD-related salivary gland involvement may be treated with saliva substitutes or sialogogues. These recommendations are based on successful treatment with pilocarpine (17) and cevimeline (18) demonstrated in open trials on samples the same size as the current study. Furthermore, the randomized controlled trial that evaluated the efficacy of pilocarpine for the treatment of cGVHD was inconclusive (19). Considering that cGVHD patients are frequently treated with numerous medications the addition of a systemic pharmacologic agent to manage dry mouth may put the patient at risk for systemic, drug related adverse events. Therefore, pharmacologic agents for salivary gland cGVHD are not an ideal treatment and the advantages of a non-pharmacological treatment delivered by an intra-oral device, with no systemic side effects, are clear.

Obviously, supportive measures to prevent damage to the teeth (e.g. rampant caries) caused by xerostomia are part of the protocol in patients with salivary gland cGVHD (15,16). These measures include frequent fluoride application and the use of a salivary stimulant (such as chewing sugar-free gum).

We planned for a larger patient enrollment in the study, however these patients are medically complex, and since dry mouth is not lethal, the low priority the patient’s placed on our trial is understandable.

In summary, this case series suggests that GenNarino is safe in oral cGVHD patients. Furthermore, in this group of patients the use of GenNarino resulted in subjective and objective improvements in dry mouth symptoms. The effects of GenNarino on systemic GVHD need to be addressed in a large-scale study.
